# Matrine Promotes Human Myeloid Leukemia Cells Apoptosis Through Warburg Effect Mediated by Hexokinase 2

**DOI:** 10.3389/fphar.2019.01069

**Published:** 2019-09-24

**Authors:** Guibin Lin, Yangzhe Wu, Fengtao Cai, Zhen Li, Shixin Su, Jian Wang, Jialin Cao, Lingdi Ma

**Affiliations:** ^1^Laboratory Center, The Third People’s Hospital of Huizhou, Affiliated Hospital of Guangzhou Medical University, Huizhou, China; ^2^The First Affiliated Hospital, Biomedical Translational Research Institute and School of Pharmacy, Jinan University, Guangzhou, China

**Keywords:** matrine, leukemia, hexokinase 2, glycolysis, apoptosis

## Abstract

Matrine, an alkaloid compound isolated from the medicinal plant *Sophora flavescens*, inhibits many types of cancer proliferation. However, the precise mechanism of the matrine antihuman chronic myeloid leukemia remains unclear. In this study, we showed that matrine significantly inhibited the cell proliferation and induced apoptosis by regulating Warburg effect through controlling hexokinases 2 (HK2) expression in myeloid leukemia cells. Interestingly, matrine inhibited the expression of HK2 mediated by reduction in c-Myc binding to HK2 gene intron and led to downregulation of HK2, which upregulated proapoptotic protein Bad and then induced apoptosis. We further demonstrated that matrine could synergize with lonidamine, an inhibitor of HK2, for the treatment of myeloid leukemia, both *in vitro* and *in vivo*. Taken together, our findings reveal that matrine could promote human myeloid leukemia cells apoptosis *via* regulating Warburg effect by controlling HK2.

## Introduction

Human myeloid leukemia, a type of malignant disease of the hematopoietic system, is due to genetic mutation and overproliferation of myeloid blasts (Ley et al., 2013; Arrigoni et al., 2018). Currently, clinical outcome of leukemia patients remains unpredictable because expected cure rates varies around 30–40% depending on gene type of leukemia. On the one hand, severe side effects and drug resistance of BCR-ABL tyrosine kinase inhibitors attenuate the clinical efficacy of chemotherapeutics (Zhang et al., 2015; Soverini et al., 2018). On the other hand, even though CD19-based chimeric antigen receptor T cells (CAR-T) has achieved great success on acute myeloid leukemia, new therapy protocols are still great demands to be developed particularly in China because of the high cost of CAR-T therapeutics. Therefore, it is imperative to develop new strategies for human myeloid leukemia treatment.

Matrine is a pleiotropic alkaloid isolated from Chinese traditional medicine radix *Sophorae flavescentis*, which has various pharmacological and physiological functions, including anti-inflammation, antivirus, antifibrosis, antioxidative, and immune regulation. In China, particularly, matrine has been clinically used to treat multiple carcinoma diseases, including hepatoma and gastric cancer (Lao, 2005; Huang et al., 2011). Previously, we demonstrated that matrine could suppress cell proliferation and induce apoptosis in human chronic myeloid leukemia (CML) cells (Ma et al., 2015). However, the underlying molecular mechanism of matrine anti-CML remains largely unclear.

It’s known that cancer cells require remarkably increased glucose consumption even in the presence of oxygen, which is named as “Warburg effect” (Warburg, 1924; Warburg et al., 1927; Warburg, 1956). Such phenomenon described that cancer cells prefer to consume glucose for energy by aerobic glycolytic, rather than mitochondrial oxidative phosphorylation. This revelation of unusual metabolism mechanism of cancer cells has been confirmed in a variety of tumor contexts and shown to associate with tumor development and progression (Han et al., 2013; Poulain et al., 2017; Lu and Hunter, 2018). For instance, impairment of glycolysis by either pyruvate kinase M2 (PKM2) or lactate dehydrogenase-A (LDHA) deletion could markedly delay leukemia initiation in both CML and acute myeloid leukemia (AML) models (Wang et al., 2014). Moreover, glycolysis suppression, by targeting rate-limiting enzymes of glycolytic pathway, may be developed into a potential way for cancer therapy (Luengo et al., 2017; Akins et al., 2018).

Hexokinases (HKs) catalyze the first committed step of glycolytic pathway by phosphorylating glucose into glucose-6-phosphate. Among four isoforms of HKs, HK1, HK2, HK3, and HK4 (also known as glucokinase) (Roberts and Miyamoto, 2015), HK2 level directly and positively correlates with glycolysis level in tumor (Warburg and Dickens, 1930). Importantly, increasing evidence support that high-level expression of HK2 is directly correlated with poor overall survival in cancer patients (Mathupala et al., 2001; Wolf et al., 2011). In contrast, HK2 depletion inhibits the tumor progression in mouse models, providing attractive prospects for tumor therapeutic strategies (Patra et al., 2013; Dewaal et al., 2018). In addition, emerging evidence showed that HK2 can depress cell apoptosis (Jiang et al., 2012; Roberts et al., 2013); however, the underlying molecular mechanism for the antiapoptotic effect of HK2 is not elucidated yet.

In this work, we found that matrine inhibited leukemia cell proliferation through cell apoptosis and glycolysis depression by regulating HK2 expression, which was mediated by proto-oncogene c-Myc. In addition, we revealed that proapoptotic protein Bad participated in promoting HK2-mediated cell apoptosis in matrine-treated cells. Furthermore, we showed that matrine could synergize with HK2 inhibitor lonidamine for confronting human myeloid leukemia. Therefore, our work provided new scientific evidences for illustrating molecular mechanism of matrine’s antihuman myeloid leukemia.

## Materials and Methods

### Cell Lines and Reagents

Human CML cell line K562 and human AML cell line HL-60 were obtained from Shanghai Cell Bank of Chinese Academy of Science (Shanghai, China) and cultured in Roswell Park Memorial Institute 1640 supplemented with 10% fetal bovine serum. Matrine was obtained from Xi’an Botanical Garden (Shanxi, China), and its purity was >99% as assessed by high-performance liquid chromatography. A stock solution was prepared in double-distilled water (ddH_2_O) at 10 mg/ml and stored at 4°C.

### Construction of Lentiviral and Stable Cell Lines

pLKO plasmid, pLVX plasmid, or control vector were cotransfected with the lentiviral packaging plasmids psPAX2 and pMD2.G into HEK293T cells for virus production. After 48 h of transfection, supernatant was collected and filtered using a 0.45-mm filter and subsequently used to infect cells. Lentiviral particles were used to directly infect K562 cells for 48 h, and then, stable clones were selected using puromycin (Invivogen, ant-pr-5b). The selected cell populations were subjected to immunoblotting to determine the silencing efficiency.

### Cell Proliferation Assay

Cells were seeded at a density of 1 × 10^5^ per well into 12-well plates, then treated with different concentrations of drugs for indicated time. Cell proliferation was assessed by cell counting or Cell Counting Kit-8 kit (Dojindo Molecular Technologies, CK04). The half-maximal inhibitory concentration (sIC50) values were determined using GraphPad Prism software through regression analysis. The concentration of drug was converted to logarithm as the X while the relative cell proliferation as the Y; then, a line chart was generated, and IC50 values were obtained.

### Validation of Drug Synergy

Synergism effect of combinational drugs was evaluated by combination index (CI) according to Chou and Talalay (1984). Cells were treated with matrine and lonidamine at four different volume ratios: 4:1, 3:2, 2:3, and 1:4. After 48 h, cell viability was measured by the Cell Counting Kit-8 assay. To calculate the IC50, the combinations of matrine and lonidamine at the four different concentration ratios were diluted 1:4 with cell culture medium into six concentration gradients. The CI was introduced to determine whether a pair of drug combinations could produce synergy. It is considered that CI < 0.9 indicates synergism, 0.9 < CI < 1.1 indicates an additive effect, and CI > 1.1 indicates antagonism.

### Metabolic Assay

The extracellular acidification rate (ECAR) was determined by a Seahorse extracellular flux (Seahorse Biosciences, XF-96) analyzer according to the manufacturer’s protocol. Briefly, cells were treated with matrine for 48 h; 5 × 10^4^ cells per well were resuspended in XF base medium (Seahorse Biosciences, 102353-100-100) with 1 mM glutamine (Sigma, G8540) and plated into XFe 96-well plates (Seahorse Biosciences, 101104-004), which were pretreated with Cell-Tak adhesive (Corning, 354240) in an environment at 37°C with a non-CO2 incubator for 1 h. After the incubation time, 10 mM glucose (Seahorse Biosciences, 9710846), 1 μM oligomycin (Seahorse Biosciences, 9710846), and 50 mM 2-dexoxy-d-glucose (2-DG) (Seahorse Biosciences, 9710846) were loaded into the injection ports in the XFe 96 sensor cartridge in sequence. Specifically, glucose was added for glycolysis assessment, and glycolytic capacity was assessed following the oligomycin injection, which inhibits oxidative phosphorylation. After injection of 2-DG, which inhibits glycolysis, nonglycolytic acidification is dominant.

### Lactate Production Assay

Lactate production was measured with a lactate assay kit (Dojindo Molecular Technologies, L256). Briefly, cells were treated with matrine for 48 h, and media on cells were replaced with phenol red-free Roswell Park Memorial Institute medium without fetal bovine serum. The plate was then incubated for 1 h at 37°C. After incubation, media from each well were assessed using the lactate assay kit. Cell numbers were counted by a microscope.

### Western Blot and Antibodies

The harvested cells were lysed with radioimmunoprecipitation assay buffer (Beyotime, P0013B) for the extraction of total protein. Protein concentration was quantified with enhanced bicinchoninic acid protein assay kit (Beyotime, P0010). Then, lysate protein was subjected to sodium dodecyl sulfate polyacrylamide gel electrophoresis and electrophoretically transferred to polyvinylidene difluoride membranes (Millipore, ISEQ00010). The membranes were sequentially blocked and incubated overnight with the following primary antibodies: HK2 (Cell Signaling Technology, 2106), platelet-type phosphofructokinase (Cell Signaling Technology, 8164), phosphoglycerate kinase 1 (Abcam, ab38007), PKM2 (Cell Signaling Technology, 4053), LDHA (Cell Signaling Technology, 3582), c-Myc (Cell Signaling Technology, 13987), B-cell lymphoma-2 (Bcl-2) (Cell Signaling Technology, 4223), Bcl-XL (Proteintech, 26967-1-AP), Bad (Cell Signaling Technology, 9239), Bax (Cell Signaling Technology, 9292) and β-actin (Sungene Biotech, KM9006). The protein bands were acquired as an electronic images format using a ChemiDocTM XRS+ System (Bio-Rad) with immobilon western chemiluminescent horseradish peroxidase substrate (Millipore, WBKLS0100) and quantified the intensities by Image Lab software.

### Real-Time PCR

Total RNA was extracted using Trizol reagent (Tiangen, DP424) and reversely transcribed into complementary DNA using PrimeScript RT reagent kit with gDNA eraser (Takara, RR047A). Transcribed complementary DNA was amplified and quantified by the real-time fluorescent quantitative PCR with a SYBR Green qPCR kit (Clontech, 639676). The relative expressions of every gene were assessed in comparison with β-actin. The sequences of the primers used were as follows: HK2 forward, 5′- GAGCCACCACTCACCCTACT-3′; HK2 reverse, 5′- CCAGGCATTCGGCAATGTG -3′; c-Myc forward, 5′-TGAGGAGACACCGCCCAC-3′; c-Myc reverse, 5′-CAACATCGATTTCTTCCTCATCTTC-3′; β-actin forward, 5′-ACGTGGACATCCGCAAAG-3′; and β-actin reverse, 5′-GACTCGTCATACTCCTGCTTG-3′.

### Chromatin Immunoprecipitation PCR

Chromatin immunoprecipitation (ChIP) assays were performed using SimpleChIP^®^ Enzymatic Chromatin IP Kit (Cell Signaling Technology, 9003) according to the manufacturer’s protocol. In brief, cells were first cross-linked by formaldehyde. Cells were then lysed, and chromatin was harvested and fragmented using enzymatic digestion and sonication to a length of approximately 150–900 base pairs. Fragmented chromatin was immunoprecipitated using rabbit anti-c-Myc antibody (Cell Signaling Technology, 13987) or rabbit immunoglobulin G (IgG) control and then extracted and purified. Two percent of precleared DNA (before addition of antibodies) was set aside as an input. Purified DNA was subjected to real-time PCR or standard PCR amplification using the primers, forward, 5′-GCCCCGCAGGTAGTCAGG-3′, and reverse, 5′-AGCCACGATTCTCTCCACG-3′.

### Flow Cytometry Analysis for Apoptosis

Cell apoptosis was detected using an apoptosis analysis kit (Sungene biotech, AO2001-02P-H) following the manufacturer’s protocol. In brief, the harvested cells were subjected to 1× binding buffer with Annexin V fluorescein isothiocyanate (FITC) and propidium iodide (PI) double-staining dye at room temperature; then, apoptotic cells were analyzed by flow cytometry (BD FACS VERSE) to be defined as those positive for Annexin V with or without PI staining.

### *In Vivo* Study

*In vivo* study was performed as previously described (Ma et al., 2017). K562 cell suspension (1 × 107 cells in 100 μl phosphate-buffered saline/mouse) was injected into the tail vein of nonobese diabetic/severe combined immunodeficiency mice at 5–6 weeks old. After 20 days of injection, mice were divided into four groups randomly. Each group was intraperitoneal injected with drugs every 2 days accordingly, while the control group was injected with phosphate-buffered saline. The mice were monitored daily and killed when they showed signs of dying. The total survival date of each group was recorded, and the survival rates were calculated by the Kaplan–Meier method.

### Statistical Analysis

Data are expressed as means ± standard deviation of the mean of separate experiments. Student’ s *t* test was applied for comparison of the means of two groups, and ANOVA was used for the means of multiple groups. Values of *P* < 0.05 were considered statistically significant.

## Results

### Matrine Suppresses Human Myeloid Leukemia Cell Proliferation and Glycolysis

**Figure 1:**
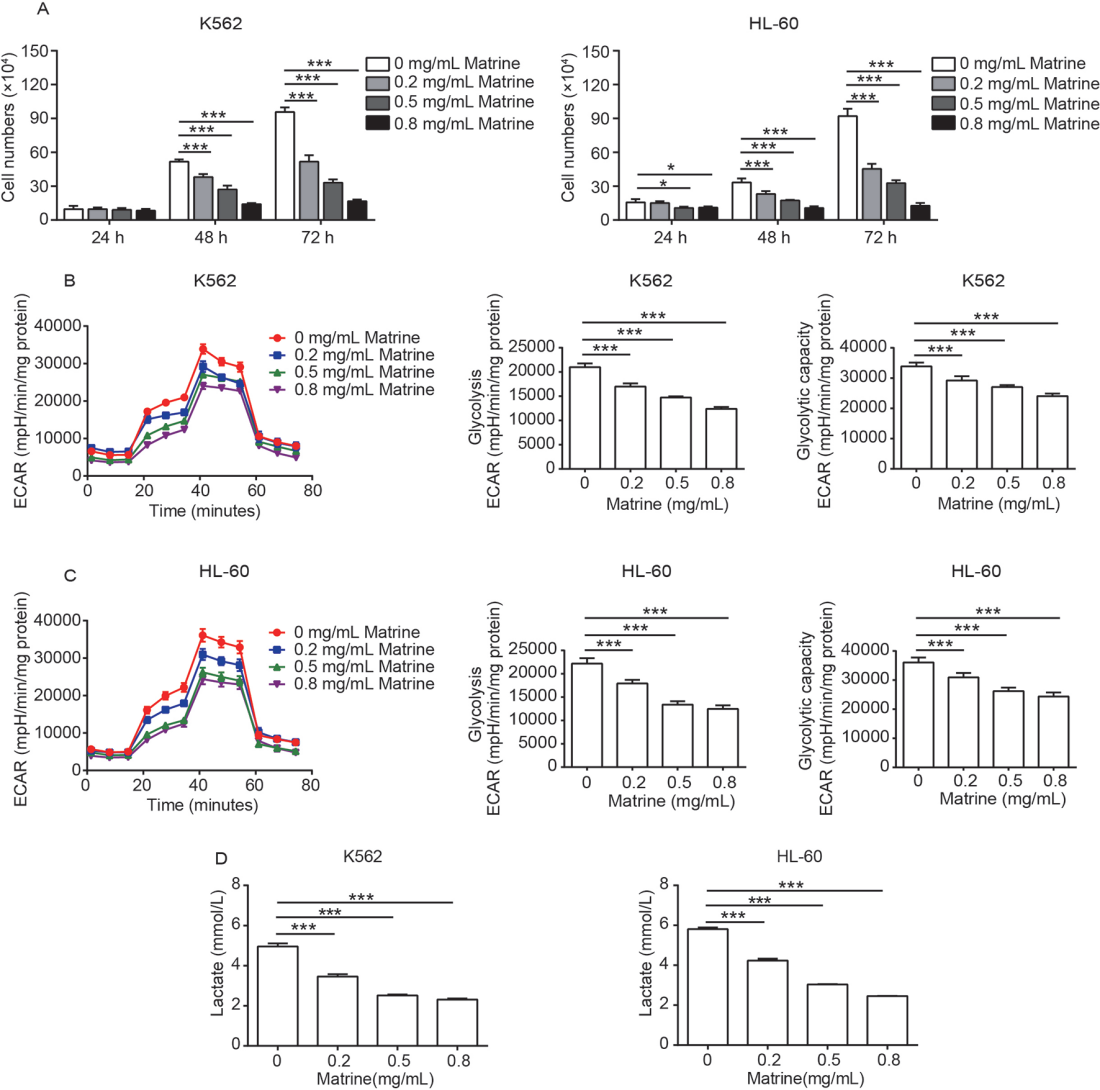
Matrine inhibits the activity of cell proliferation and glycolysis in human myeloid leukemia cells. K562 and HL-60 cells were treated with different concentrations of matrine for 24, 48, and 72 h, and cell numbers were measured by cell counting **(A)**. The glycolysis, glycolysis capacity, and lactate production of K562 and HL-60 cells were measured by extracellular acidification rate and lactate assay kit **(B–D)**, respectively, following the indicated concentrations of matrine treatment for 48 h. Data were mean ± SD (*n* = 3). *P < 0.05, ***P < 0.001.

To determine the effect of matrine on the proliferation of human myeloid leukemia cells, we treated human CML cell line K562 and human AML cell line HL-60 with different concentrations of matrine, and cell viability was measured. Our data showed that matrine effectively inhibited the proliferation of K562 and HL-60 cells in a dose- and time-dependent manner. The IC50 values for 48 h was ∼0.5 mg/ml in both K562 and HL-60 cells (**Figure 1A** and **Supplementary Figure 1A**).

Reprogramming glucose metabolism is considered as a hallmark of cancer cells (Hanahan and Weinberg, 2011), and previous works reported energy metabolic disturbance of leukemia cells including increased glycolysis, higher glucose uptake, and higher lactic acid production (Boag et al., 2006; Jitschin et al., 2015). To assess whether glycolysis is involved in matrine-induced leukemia cell growth inhibition, we measured the ECAR of matrine-treated K562 and HL-60 cells for 48 h. As presented in **Figures 1B, C**, compared with the control group, matrine treatment could significantly suppress both glycolysis and the glycolytic capacity in a dose-dependent manner. We further observed that matrine dramatically decreased the lactate production in both K562 and HL-60 cells in a dose-dependent manner (**Figure 1D**). These data are accordant with cell viability assessment, implicating that glycolysis plays an important role in matrine inhibiting the proliferation of human myeloid leukemia cells.

### Matrine Downregulates HK2 Expression Through C-Myc Inhibition

**Figure 2:**
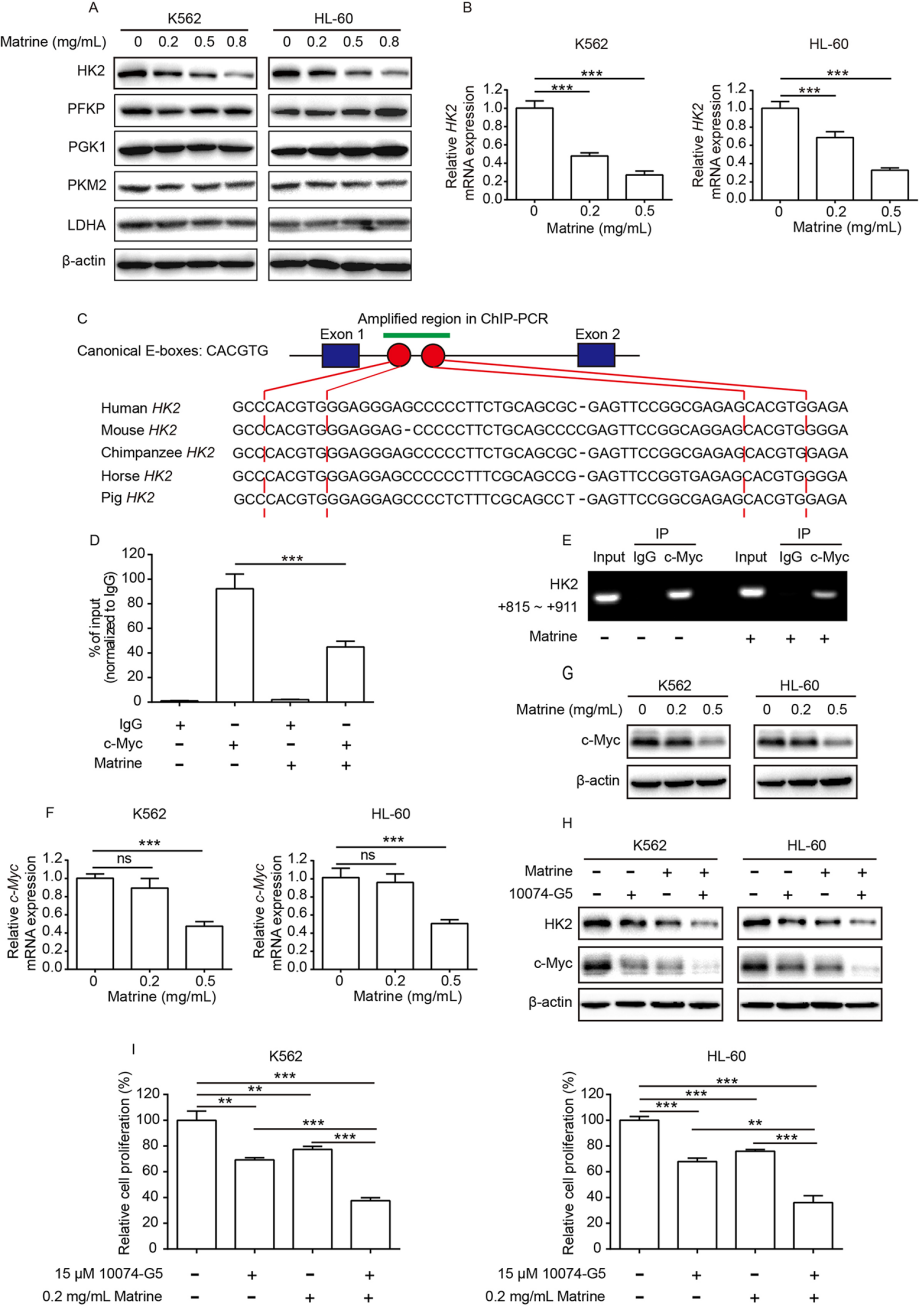
c-Myc is important for matrine-induced downregulation of HK2. K562 and HL-60 cells were treated with indicated concentrations of matrine for 48 h, and a number of key metabolic enzyme involved in glycolysis expression were measured by Western blot **(A)**, and *HK2* mRNA expression was measured by real-time PCR **(B)**. Schematic representation of canonical E-boxes, which are c-Myc-binding elements, localize in the first intron of *HK2* gene. Sequence alignment of human HK2 with the corresponding region in mouse, chimpanzee, horse, and pig **(C)**. ChIP assay was performed with anti-c-Myc antibody or IgG in K562 cells treated with or without matrine; then, purified DNA was subjected to real-time PCR **(D)** or standard PCR analysis **(E)**. K562 and HL-60 were treated with indicated concentrations of matrine for 48 h; *c-Myc* mRNA and c-Myc protein expression were measured by real-time PCR **(F)** and Western blot **(G)**, respectively. Western blot analysis of HK2 and c-Myc protein expression in K562 and HL-60 cells treated with matrine (0.5 mg/ml) or 10074-G5 (15 μM) alone or in combination for 48 h **(H)**. K562 and HL-60 cells were treated with matrine (0.2 mg/ml) or 10074-G5 (15 μM) alone or in combination for 48 h, and relative cell proliferation was measured by cell counting **(I)**. Data were mean ± SD (*n* = 3). **P < 0.01, ***P < 0.001.

To probe the molecular mechanism of how matrine depresses glycolysis of K562 and HL-60 cells, we then examined the expression of a number of key metabolic enzymes involved in glycolysis, including HK2, platelet-type phosphofructokinase, phosphoglycerate kinase 1, PKM2, and LDHA. We performed Western blot analyses and found that HK2 protein expression level was significantly downregulated by matrine in a dose-dependent manner. The expression of other key enzymes was not affected by matrine, except that PKM2 and LDHA were slightly downregulated by high concentration of matrine (**Figure 2A**, **Supplementary Figure 2A**). We also analyzed the effect of matrine on *HK2* messenger RNA (mRNA), and the data showed that matrine could significantly reduce *HK2* mRNA expression in a dose-dependent manner (**Figure 2B**).

Recent studies showed that c-Myc binds to the regulatory region of the *HK2* gene and plays a pivotal role in glucose metabolism (Kim et al., 2004; Dejure and Eilers, 2017). Through sequence alignment, we identified two canonical c-Myc-binding sites (E-boxes) in the first intron of the *HK2* gene of different species, including human, mouse, chimpanzee, horse, and pig. High similarity of c-Myc-binding sites in the *HK2* gene implies that c-Myc-binding sites are highly conserved across species (**Figure 2C**). By performing ChIP assay with anti-c-Myc antibody or anti-IgG antibody, we verified that c-Myc binds to the first intron of the *HK2* gene (left graph in **Figures 2D, E**). Thus, it is important to explore whether or not c-Myc is involved in matrine-induced depression of *HK2* transcription. We found that the amount of c-Myc binding to *HK2* gene was strikingly decreased upon matrine treatment (right graph in **Figures 2D, E**). To further verify the effects of matrine on c-Myc, real-time PCR and Western blot analysis were performed. As shown in **Figures 2F, G**, 0.5 mg/ml matrine displayed strong inhibition on both *c-Myc* mRNA and c-Myc protein expression. In addition, further experiments showed that c-Myc inhibitor 10074-G5 enhanced the inhibitory effect of matrine on c-Myc and HK2 expression, and cell proliferation in both K562 and HL-60 cells (**Figures 2H, I** and **Supplementary Figure 1B**). Collectively, these results support that c-Myc plays key role in matrine-induced downregulation of HK2.

### HK2 Displays an Antagonistic Role in Matrine-Induced Cell Apoptosis

**Figure 3:**
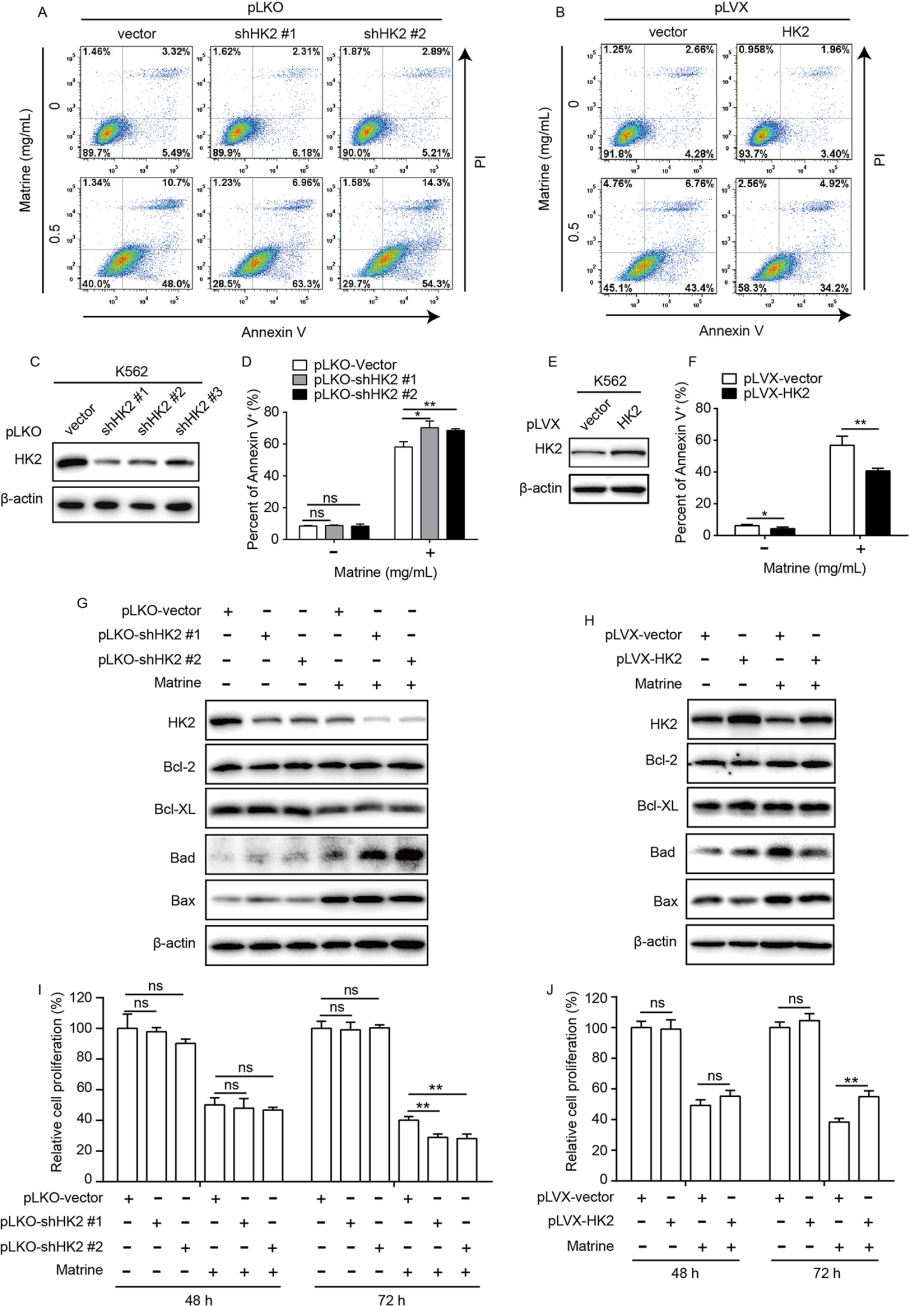
HK2 antagonized matrine-induced human myeloid leukemia cell apoptosis. K562 cells were infected with lentivirus-based short hairpin RNAs **(C)** or overexpression **(E)** vector for 48 h and selected by puromycin (5 μg/ml) for 5 days; then, HK2 protein expression were measured by Western blot, respectively. K562 cells with HK2 stable knockdown were treated with or without matrine (0.5 mg/ml) for 48 h; cell apoptosis was analyzed by flow cytometry using AnnexinV/PI double staining **(A, D)**, and Bcl-2 family members expression was analyzed by Western blot **(G)**. K562 cells with HK2 overexpression were treated with or without matrine (0.5 mg/ml) for 48 h, cell apoptosis was analyzed by flow cytometry using AnnexinV/PI double staining **(B, F)**, and Bcl-2 family members expression was analyzed by Western blot **(H)**. HK2 stable knockdown **(I)** or overexpression **(J)** K562 cells were treated with or without matrine (0.5 mg/ml) for 48 and 72 h; relative cell proliferation was measured by cell counting. Data were mean ± SD (*n* = 3). *P < 0.05, **P < 0.01.

In addition to suppressing glycolysis, we also confirmed that matrine induced apoptosis in K562 cells (**Figures 3A, B**). To explore the role of HK2 in matrine-induced cell apoptosis, we used three lentivirus-based short hairpin RNAs against HK2 and infected K562 cells for stable cell lines after selection. It showed that all short hairpin RNAs knockdown HK2 efficiently, particularly shHK2 #1 and shHK2 #2 (**Figure 3C**). Subsequent experiments were carried out using shHK2 #1 and shHK2 #2 stable cell lines. K562 stable cell lines were treated with or without matrine for 48 h and then analyzed by flow cytometry with FITC-conjugated Annexin V and PI. Here, our data showed that, compared with the pLKO vector, HK2 depletion did not influence K562 cell apoptosis itself in the absence of matrine. We found that treatment with 0.5 mg/ml matrine elevated the proportion of Annexin V-positive apoptotic cells in HK2 knockdown cells when compared with the pLKO vector (**Figures 3A, D**). To further confirm the role of HK2, we constructed HK2 overexpression K562 cell line as well (**Figure 3E**). As expected, HK2 overexpression significantly inhibited matrine-induced cell apoptosis compared with the pLVX vector (**Figures 3B, F**). Therefore, these data suggest that HK2 displays an antagonistic effect in matrine-induced apoptosis.

To further detect the mechanism by which HK2 antagonized matrine-induced apoptosis, we tested whether or not Bcl-2 family genes are involved because Bcl-2 genes are critical in apoptotic signaling pathway associated with environmental stress signals. To this end, we checked the expression of Bcl-2 family members including pro- and antiapoptotic proteins by Western blot assay. As shown in **Figure 3G**, matrine suppressed the expression of the antiapoptotic proteins Bcl-XL, but not Bcl-2, while the expression of the proapoptotic proteins Bad and Bax was upregulated. Surprisingly, HK2 knockdown significantly enhanced matrine-induced upregulation of Bad protein expression. On the contrary, HK2 overexpression attenuated the upregulation of Bad expression induced by matrine (**Figure 3H**). Therefore, these results suggest that HK2 antagonized matrine-induced apoptosis *via* regulating proapoptotic protein Bad expression.

In addition, we further showed that knockdown or overexpression of HK2 has no detected adverse effect on K562 cell proliferation. However, HK2 knockdown could enhance the inhibitory effect of matrine on the proliferation of K562 cells, while HK2 overexpression had an opposite effect (**Figures 3I, J** and **Supplementary Figure 1C**), suggesting that HK2 is crucial for matrine-mediated cytotoxicity of K562 cells.

### Matrine Synergizes With Lonidamine in Human Myeloid Leukemia Cells

**Figure 4:**
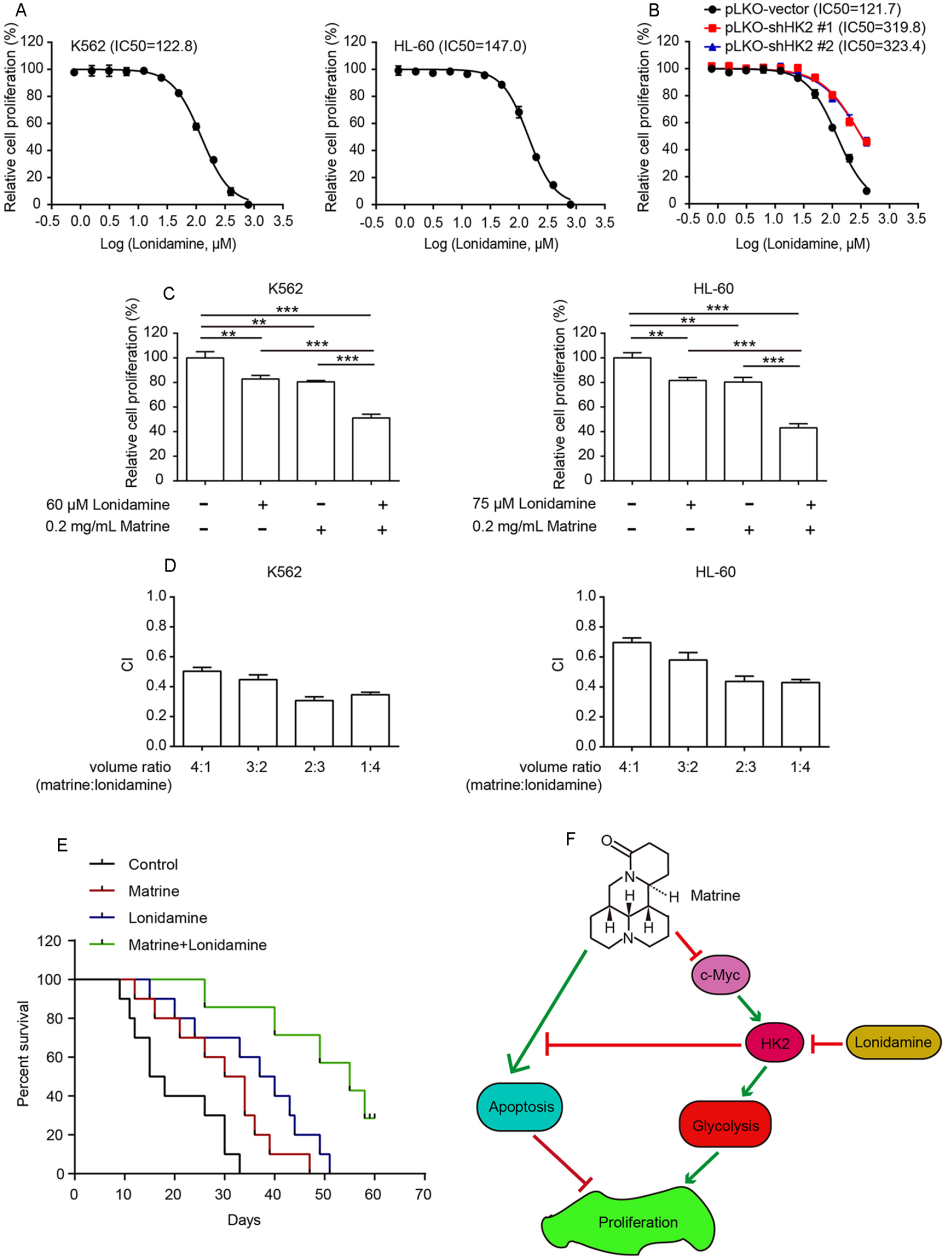
Combining matrine with lonidamine exhibits synergistic effect in human myeloid leukemia cells. K562 and HL-60 cells were treated with different concentrations of matrine for 48 h, and relative cell proliferation was measured by cell counting **(A)**. K562 cells with HK2 stable knockdown were treated with different concentrations of matrine for 48 h, and relative cell proliferation was measured by cell counting **(B)**. K562 and HL-60 cells were treated with matrine or lonidamine alone or in combination for 48 h, and relative cell proliferation was measured by cell counting **(C)**. K562 and HL-a60 cells were treated with a serial volume ratio combining matrine and lonidamine for 48 h; synergistic effect of matrine and lonidamine was assessed according to approach described by Chou and Talalay. The combination index (CI) < 0.9 indicates synergism **(D)**. K562-cell-bearing mice were divided into four groups randomly, and each group was intraperitoneally injected with matrine (20 mg/kg) or lonidamine (30 mg/kg) or in combination every 2 days accordingly. Kaplan–Meier analysis of survival rates of each group until day 60 **(E)**. Working model of antihuman myeloid leukemia cells by combination treatment with matrine and lonidamine **(F)**. **P < 0.01, ***P < 0.001.

Given that knockdown of HK2 promotes matrine-induced cell apoptosis and growth inhibition, we examined whether or not pharmacological inhibition of HK2 could sensitize human myeloid leukemia cells to matrine challenge. Lonidamine, a pharmacological inhibitor of HK2, can effectively suppress multifarious cancer cell proliferation and metastasis *in vitro*, *in vivo*, as well as clinical trials (Floridi et al., 1981; Di Cosimo et al., 2003; Thamrongwaranggoon et al., 2017), which is thus used in our experiment. First, we measured relative cell proliferation of K562 and HL-60 cells treated with lonidamine and found that the concentration of lonidamine-inhibited cell proliferation at IC50 values is approximately 122.8 and 147.0 μM for K562 and HL-60 cells, respectively (**Figure 4A**). We also confirmed that lonidamine suppresses cell growth by targeting HK2. Knockdown of HK2 could reduce the sensitivity of K562 cells to lonidamine compared to the pLKO vector (**Figure 4B**).

Next, K562 and HL-60 cells were incubated with a combination of matrine and lonidamine at low concentration below the IC50. As shown in **Figure 4C** and **Supplementary Figure 1D**, cotreatment of K562 cells with matrine and lonidamine exhibited significant inhibitory effect on cell growth compared with matrine or lonidamine alone. Similar results were obtained in HL-60 cells as well (right graph in **Figure 4C** and **Supplementary Figure 1D**). We then tested the synergistic effect of matrine and lonidamine by assessing CI according to Chou and Talalay. Synergetic effect (CI < 0.9) was observed between the combination of matrine and lonidamine in both K562 and HL-60 cells (**Figure 4D**). Furthermore, we examined this synergistic effect *in vivo*. As expected, Kaplan–Meier curves showed that cotreatment with matrine and lonidamine evidently prolonged the survival of mice bearing K562 cells compared with matrine or lonidamine alone (**Figure 4E**). Taken together, these results suggest that matrine can synergize with pharmacological inhibitor of HK2 against human myeloid leukemia cells.

## Discussion

For normal cells, glucose is metabolized by the coupling of glycolysis to the tricarboxylic acid cycle. Contrarily, cancer cells reprogram cellular glucose metabolism to fulfill excessive biosynthetic demands of cell proliferation in tumor microenvironment. Regardless of oxygen concentration, cancer cells tend to utilize glucose metabolism *via* the glycolytic pathway instead of the tricarboxylic acid cycle. Such reprogramming of glucose metabolism benefits both bioenergetics and biosynthesis necessary for cell growth and division. High glycolytic rate allows cells to use glucose to produce abundant adenosine 5′-triphosphate. In addition, increased glycolysis promotes diversion of glycolytic intermediates into various biosynthetic pathways, including ribose sugars for nucleotides, glycerol, and citrate for lipids, nonessential amino acids, as well as nicotinamide adenine dinucleotide phosphate (Vander Heiden et al., 2009; Ward and Thompson, 2012). Such reprogrammed glucose metabolism happens in leukemia cells as well (Boag et al., 2006; Jitschin et al., 2015). Therefore, inhibition of glycolysis by targeting key enzymes of glycolytic pathway is considered as a potential therapeutic approach for leukemia.

In the present study, we found that matrine could significantly inhibit both glycolysis, glycolytic capacity, and lactate production in human myeloid leukemia cell lines K562 and HL-60. Moreover, we found that matrine could inhibit the expression of c-Myc and HK2, which are a transcription regulator reprogramming tumor cell metabolism and a glycolytic rate-limiting enzyme catalyzing the phosphorylation of glucose to produce glucose-6-phosphate, respectively (Roberts and Miyamoto, 2015; Dejure and Eilers, 2017). By ChIP assay, we showed that matrine dramatically reduced c-Myc to bind with *HK2* gene. These data suggested that inhibition of c-Myc by matrine leads to downregulation of HK2, thereby depressing glycolysis in human myeloid leukemia cells.

It has been shown that HK2 is a key rate-limiting enzyme for glycolysis, and our work demonstrated that HK2 can act as an antagonist in matrine-induced apoptosis. This can be supported by flow cytometry with FITC-conjugated Annexin V and PI that HK2 overexpression could promote matrine-induced apoptosis in K562 cells. To further explore the mechanism of HK2-mediated apoptosis upon matrine treatment, we examined the protein expression of Bcl-2 family, including Bcl-2, Bcl-XL, Bad, and Bax. We found that expression of proapoptosis protein Bad is inversely correlated with HK2 expression level in K562 stable cell lines treated with matrine, suggesting that HK2 exerts antagonistic effect on matrine-induced apoptosis by regulating proapoptotic protein bad expression.

In addition, lonidamine, a derivative of indazole-3-carboxylic acid, can restrict cancer cell glucose metabolism by targeting HK2. As a clinically used antitumor drug, lonidamine’s clinical efficacy is still obscure (Di Cosimo et al., 2003; Thamrongwaranggoon et al., 2017). However, combination with certain chemotherapy such as paclitaxel and epirubicin can greatly improve the therapeutic efficacy of lonidamine (Dogliotti et al., 1996; De Lena et al., 2001). Here, we combined matrine with lonidamine and found that inhibition of HK2 by lonidamine promotes the antihuman myeloid leukemia cell effect of matrine, both *in vitro* and *in vivo*. This proposed a new strategy in clinical application to enhance efficacy of lonidamine by combining with matrine.

Collectively, our work revealed that matrine inhibits human myeloid leukemia cell proliferation though suppressing glycolysis and inducing cell apoptosis. Specifically, matrine depresses glycolysis by downregulating HK2 expression *via* inhibiting transcription regulator c-Myc activity. Meanwhile, HK2 participates in regulating Bad protein expression to antagonize matrine-induced apoptosis. Our study further revealed that matrine plays a synergistic effect to enhance lonidamine efficiency for leukemia treatment (**Figure 4F**). Altogether, our present work provided new scientific evidences for illustrating molecular mechanism of matrine’s antihuman myeloid leukemia and proposed a new strategy for the clinical treatment of leukemia.

## Data Availability

The raw data supporting the conclusions of this manuscript will be made available by the authors, without undue reservation, to any qualified researcher

## Ethics Statement

Ethical approval for the study was granted by the Ethics Committee of Dalian Medical University.

## Author Contributions

GL and ZL performed and analyzed all the experiments. SS, JW, JC, and FC drafted the work for important intellectual content. YW edited the language and figures. LM wrote the manuscript and designed the study.

## Funding

This work was supported by the National Nature Science Foundation of China (81673644), Medical Scientific Research Foundation of Guangdong Province (A2016252), Research Projects of Guangdong Provincial Bureau of Traditional Chinese Medicine (20181261), Science and Technology Plan (Medical and Health) Project of Huizhou (2018Y158), Scientific Research Team Construction Project of Huizhou Third People’s Hospital (2016A001), and partially supported by the “111 project” (B16021).

## Conflict of Interest Statement

The authors declare that the research was conducted in the absence of any commercial or financial relationships that could be construed as a potential conflict of interest.

## Supplementary Materials

Figure S1 K562 and HL-60 cells were treated with different concentrations of matrine for 24, 48, and 72 h, and relative cell proliferation was measured by CCK-8 assay **(A)**. K562 and HL-60 cells were treated with matrine (0.2 mg/ml) or 10074-G5 (15 μM) alone or in combination for 48 h, and relative cell proliferation was measured by CCK-8 assay **(B)**. HK2 stable knockdown or overexpression K562 cells were treated with or without matrine (0.5 mg/mL) for 48 and 72 h, relative cell proliferation was measured by CCK-8 assay **(C)**. K562 and HL-60 cells were treated with matrine or lonidamine alone or in combination for 48 h, and relative cell proliferation was measured by CCK-8 assay **(D)**. Data were mean ±SD (n = 3). **P < 0.01, ***P < 0.001.

Figure S2 K562 and HL-60 cells were treated with indicated concentrations of matrine for 48 h, and the protein expression of HK2, PFKP, PGK1, PKM2 and LDHA were measured by Western blot, then the protein bands’ intensities was quantified by Image Lab software **(A)**. Data were mean ±SD (n = 3). *P < 0.05, ***P < 0.001.
