# Supplementary figures and images for: Matrine Promotes Human Myeloid Leukemia Cells Apoptosis Through Warburg Effect Mediated by Hexokinase 2
Source: Front Pharmacol. 2019 Sep 24;10:1069. doi: 10.3389/fphar.2019.01069 (PMC6771294; doi:10.3389/fphar.2019.01069)

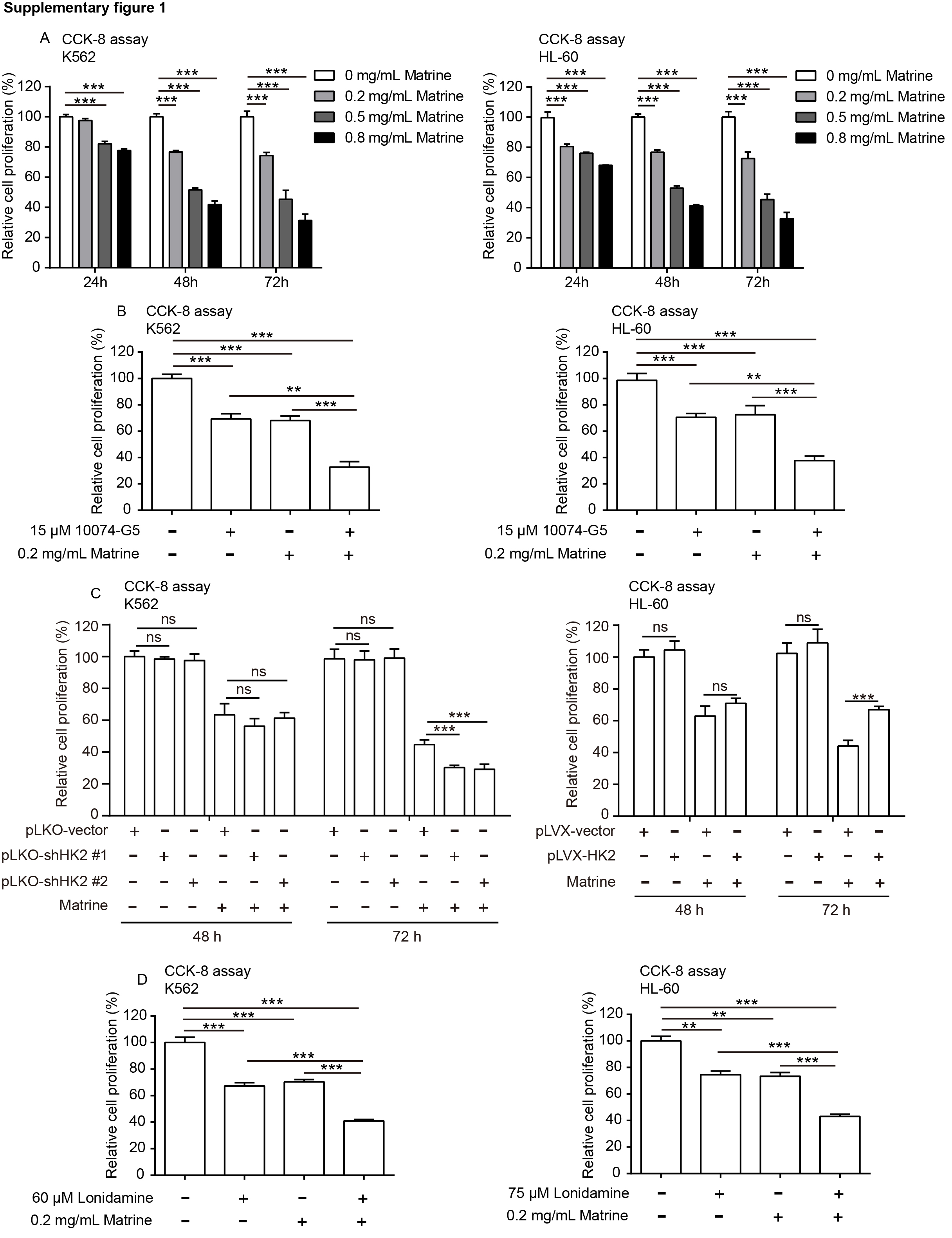

Supplement: Figure S1 — K562 and HL-60 cells were treated with different concentrations of matrine for 24, 48, and 72 h, and relative cell proliferation was measured by CCK-8 assay (A). K562 and HL-60 cells were treated with matrine (0.2 mg/ml) or 10074-G5 (15 μM) alone or in combination for 48 h, and relative cell proliferation was measured by CCK-8 assay (B). HK2 stable knockdown or overexpression K562 cells were treated with or without matrine (0.5 mg/mL) for 48 and 72 h, relative cell proliferation was measured by CCK-8 assay (C). K562 and HL-60 cells were treated with matrine or lonidamine alone or in combination for 48 h, and relative cell proliferation was measured by CCK-8 assay (D). Data were mean ±SD (n = 3). **P < 0.01, ***P < 0.001. [file Image_1.jpeg]

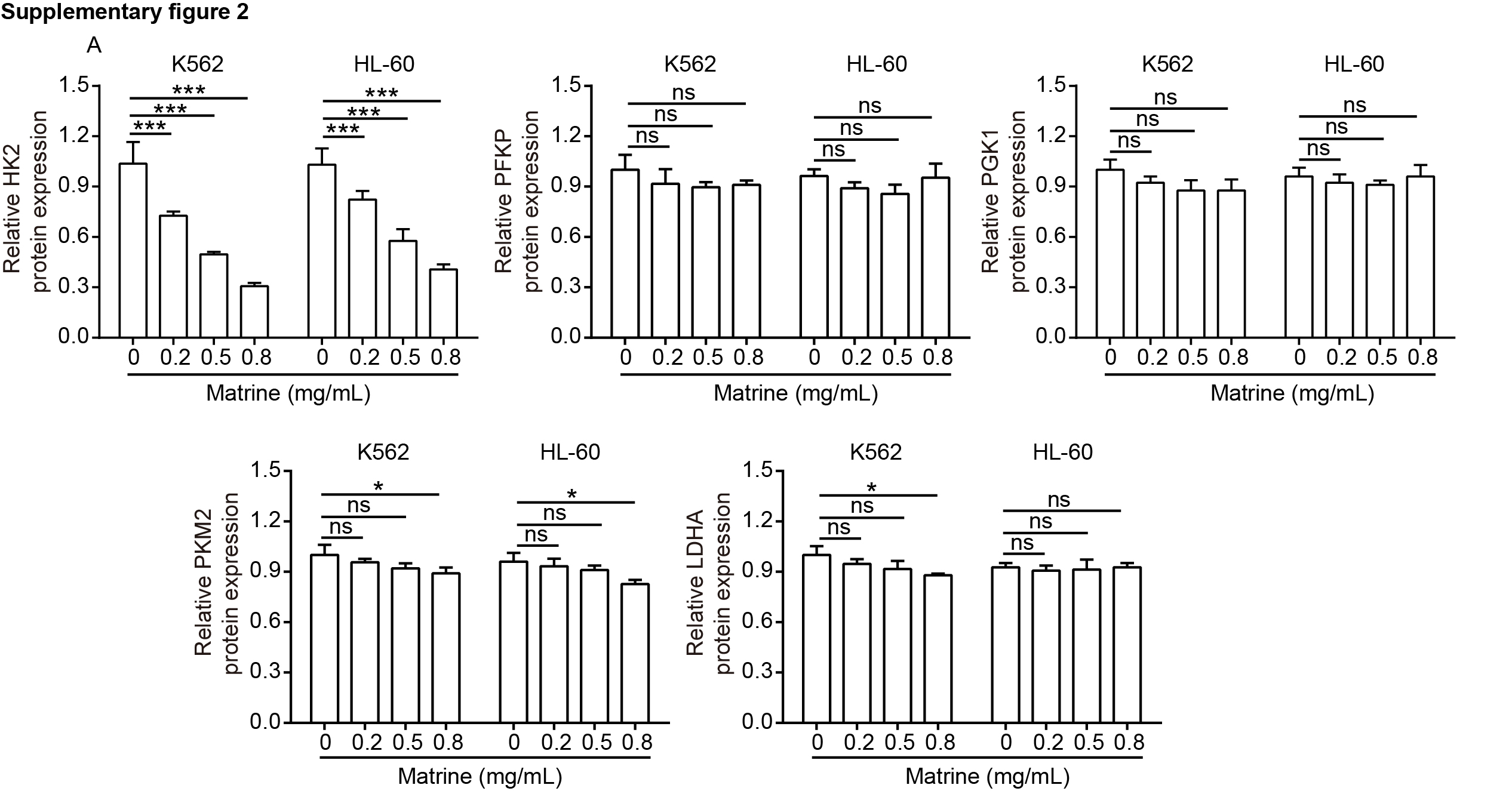

Supplement: Figure S2 — K562 and HL-60 cells were treated with indicated concentrations of matrine for 48 h, and the protein expression of HK2, PFKP, PGK1, PKM2 and LDHA were measured by Western blot, then the protein bands’ intensities was quantified by Image Lab software (A). Data were mean ±SD (n = 3). *P < 0.05, ***P < 0.001. [file Image_2.jpeg]
